# Lipid metabolism in cancer cells under metabolic stress

**DOI:** 10.1038/s41416-019-0451-4

**Published:** 2019-05-16

**Authors:** Rimsha Munir, Jan Lisec, Johannes V. Swinnen, Nousheen Zaidi

**Affiliations:** 1 Cancer Biology Lab, MMG, University of the Punjab, Lahore, 54590 Pakistan; 2 Bundesanstalt für Materialforschung und -prüfung (BAM), Department of Analytical Chemistry, Richard-Willstätter-Straße 11, 12489 Berlin, Germany; 3 Laboratory of Lipid Metabolism and Cancer, Department of Oncology, Faculty of Medicine, KU Leuven, Leuven, Belgium

**Keywords:** Cancer microenvironment, Cancer metabolism

## Abstract

Cancer cells are often exposed to a metabolically challenging environment with scarce availability of oxygen and nutrients. This metabolic stress leads to changes in the balance between the endogenous synthesis and exogenous uptake of fatty acids, which are needed by cells for membrane biogenesis, energy production and protein modification. Alterations in lipid metabolism and, consequently, lipid composition have important therapeutic implications, as they affect the survival, membrane dynamics and therapy response of cancer cells. In this article, we provide an overview of recent insights into the regulation of lipid metabolism in cancer cells under metabolic stress and discuss how this metabolic adaptation helps cancer cells thrive in a harsh tumour microenvironment.

## Background

The development and progression of cancer is typically accompanied by marked changes in the tumour microenvironment. The rapid growth and expansion of tumour tissue often leads to a poor and aberrant blood supply, resulting in hypoxia and a limited supply of nutrients. To thrive under these changing and challenging conditions, cancer cells adapt their metabolism, including that of lipids.^1–3^ One of the key features of this metabolic adaptation is the elevated de novo synthesis of fatty acids (FAs), which is observed in many different cancer types^4–8^ and is generally believed to be required to provide rapidly proliferating cancer cells with a constant supply of FAs for membrane biogenesis, energy production and protein modification. Fatty acid synthase (FASN), the rate-limiting enzyme in the FA synthesis pathway, has been widely reported to promote cancer progression.^3^ However, studies have shown that cancer cells can also acquire exogenous FAs by upregulating various FA-uptake mechanisms.^9–11^

Several factors, including genetic mutations, play a significant role in determining the relative dependence of cancer cells on lipid uptake versus endogenous synthesis. For instance, cells transformed by oncogenic HRAS^G12V^ display elevated lipid uptake, whereas cells transformed by constitutively active (myristoylated) AKT undergo increased de novo synthesis.^9^ In addition, crosstalk between tumour cells and the microenvironment affects the cellular lipid acquisition mode. Oxygen and nutrient deprivation can each independently induce metabolic stress, which affects the balance between FA synthesis and uptake,^3,9^ and consequently the lipid composition of cancer cells.^12–14^ This metabolic flexibility is particularly important for cancer cells within tumour tissues that are often exposed to temporal fluctuations in oxygen and nutrient availability.

This review discusses the effects of oxygen and nutrient deprivation, as separate parameters and in combination, on various aspects of lipid metabolism in cancer cells. We have performed an extensive literature survey, and, through an in-depth analysis of the results disseminated throughout the published literature, tried to explain the inconsistencies in reports and to link these inconsistencies to different experimental/analytical methods, including differences in cell line models, cell culture conditions or data presentation strategies. Therefore, in this review we have focused not only on summarising the major findings but also on detailing the methodological variations in previous reports, which are provided as supplemental information. The findings discussed in this review reveal that oxygen and nutrient deprivation limit cancer cells’ metabolic flexibility that otherwise allows these cells to switch between different pathways of FA acquisition. Hence, under such conditions the pathway currently adopted by the cancer cell could be the preferred target of anti-neoplastic strategies. Otherwise, therapeutic strategies simultaneously targeting several routes of lipid provision might be required.

### Lipid metabolism under hypoxic conditions

Hypoxia is a common feature of many human tumours and is a consequence of high cell proliferation rates, resulting in increased oxygen consumption, and aberrant blood vessel development. Hypoxia promotes aggressive malignancy and is associated with poor prognosis in a wide range of cancer types.^15–17^ Oxygen deprivation results in the expression of hypoxia-inducible factors (HIFs), which mediate multiple protective mechanisms that help in maintaining oxygen homoeostasis by reducing oxidative metabolism and oxygen consumption.^18–21^ One such inducible factor, HIF-1α, is a key regulator of multiple cancer-related metabolic pathways, including glycolysis, glycogenesis, the TCA cycle, nucleotide metabolism, amino acid metabolism, leptin metabolism, lipid metabolism and others.^22–25^

### FA synthesis under hypoxic conditions

**Fig. 1:**
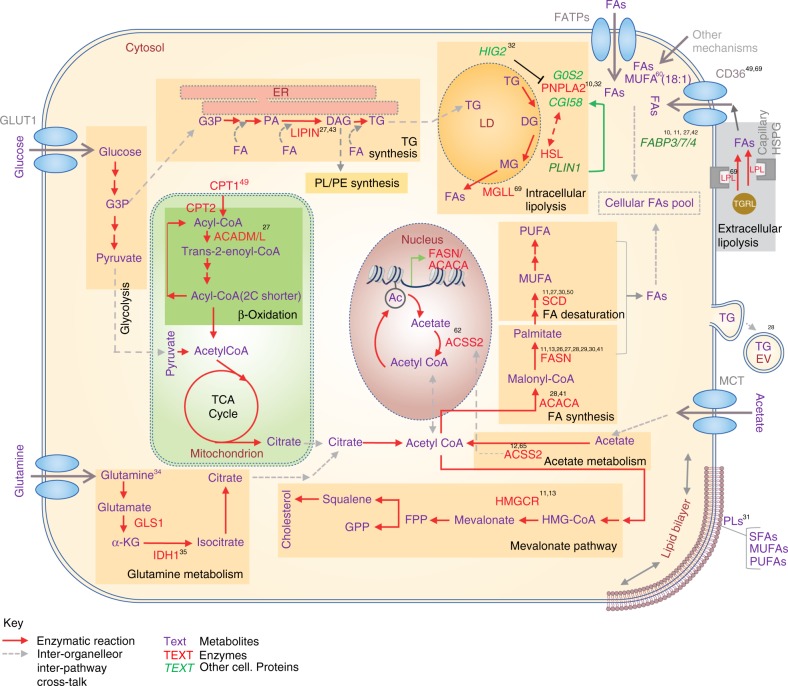
Overview of the major lipid metabolism pathways shown to be affected by metabolic stress. The figure highlights all the key lipid metabolism pathways activated in cancer cells. Major pathways are shown as boxes without outlines. The systematic names of these pathways are given at the bottom-right corner of each box. The numbers shown superscripted to each protein/metabolite indicate the reference number. For further details see the text and Supplementary Table 1. ACACA acetyl-CoA carboxylase 1, ACACB acetyl-CoA carboxylase 2, ACSS2 acyl Co-A synthetase-2, ADFP adipose differentiation protein, ATGL adipose triglyceride lipase, FA fatty acids, FABP3 fatty acid binding protein 3, FABP7 fatty acid binding protein 7, FFA free fatty acids, FASN fatty acid synthase, H hypoxia, HIF-1α hypoxia-inducible factor 1-α, HIF-2α hypoxia-inducible factor 2α, HIG2 hypoxia-inducible gene 2 protein, HMGCR 3-hydroxy-3-methylglutaryl-CoA reductase, LD lipid droplet, LCAD long-chain specific acyl-CoA dehydrogenase, MAG monoacylglycerol, MCAD medium-chain acyl-CoA dehydrogenase, MUFA monounsaturated fatty acids, PBMCs peripheral blood mononuclear cells, PC phosphatidylcholines, PE phosphatidylethanolamines, Pcho propargyl-choline, PI phosphatidylinositol, PS phosphatidylserine, PUFA polyunsaturated fatty acids, SCD stearoyl-CoA desaturase, SFA saturated fatty acids, SREBP sterol regulatory element-binding proteins, TG triglycerides

Several groups have studied the effects of hypoxic stress on FA synthesis in cancer cells, but the results of these studies appear to be largely inconsistent. For instance, FASN expression has been reported to be increased,^26–28^ decreased^11,29^ or unaffected^30^ in hypoxic cancer cells (Table 1, Fig. 1; FA Synthesis). The most obvious differences among these studies come from the selection of cancer type or cell line models (Table 1), suggesting the potential existence of a cell-type-specific regulation of FA synthesis under hypoxia. However, detailed examination of the previous reports revealed other subtle to substantial differences in cell culture methods (Supplementary Table 1), which might also induce variations in the results. For instance, in some studies, cells were serum-starved prior to hypoxia induction,^31^ hypoxia was applied in combination with nutrient deprivation,^12^ or full serum media was supplemented with exogenous lipids.^32^ Indeed, Lewis et al.^11^ reported that, in glial cancer cells, hypoxia alone led to the decreased expression of FA synthesis markers, while hypoxia in combination with low-serum conditions led to an increased expression of these markers. Interestingly, hypoxic regions within tumours growing in vivo display increased FASN expression (Supplementary Table 1).^26^ Hence, we can speculate that cells in these hypoxic cores are simultaneously deprived of oxygen and nutrients. Further studies are required to prove this hypothesis under in vivo settings. In addition, FASN expression under hypoxic conditions seems to be dependent on cell seeding density. FASN expression was downregulated in high-density HepG2 human hepatoblastoma cultures, while it remained unaffected in low-density cell cultures.^29^

**Table 1:** Regulation of lipid metabolism under metabolic stress

Stress	Cancer type	Observed effects on lipid metabolism	Reference
H	Breast	FASN expression upregulated via activation of Akt and SREBP-1	^26^
H	Liver, prostate	Expression of markers of FA synthesis (FASN), FA desaturation (SCD-1) and TG synthesis (LIPIN1) upregulated; expression of markers of FA β-oxidation (ACADM and ACADL) and FA uptake (FABP7) downregulated; cellular TG levels increased	^27^
H	Liver	FASN expression downregulated only in high-density cell cultures, where hypoxia induced cytotoxic effects; FASN expression unaffected in low-density cell cultures	^29^
H	Brain	Expression of markers of FA synthesis (FASN, ACACA and ACACB) and mevalonate synthesis (HMGCR) downregulated; expression of markers of FA desaturation (SCD-1) and FA uptake (FABP3 and 7) upregulated	^11^
H	Colorectal	Expression of FA synthesis (FASN and ACACA) markers either unaffected or downregulated; expression of FA desaturation (SCD-1) markers upregulated	^30^
H	Liver, breast, prostate	Under hypoxia, acetate also functions as an epigenetic metabolite that enhances H3 acetylation levels in FASN and ACACA promoter regions, which upregulates FASN and ACACA expression, and increases FA synthesis	^41^
H	Breast, brain	Cancer cells accumulated lipid droplets under hypoxia through FA uptake (via upregulated FABP3/7), while de novo FA synthesis was repressed. Expression of perilipin 2 (a protein in lipid droplet membranes) was also upregulated. Cellular TG levels increased and TG profiles were differentially affected in different cancer cell lines	^10^
H	Breast, cervical, lung	Cancer cells showed increased FA [particularly of MUFA (C18:1)] uptake. Glutamine was the primary carbon source for synthesis of acetyl-CoA	^9^
H	Renal, colorectal	Intracellular lipolysis suppressed due to inhibition of PNPLA2 by HIG2, causing increased TG levels in hypoxic cells	^32^
H	Breast, cervical, lung	Cancer cells mainly reliant on glutamine and acetate for the synthesis of acetyl-CoA	^34^
H	Lung, breast, skin, colorectal	Under hypoxic conditions, reductive carboxylation of glutamine-derived α-ketoglutarate (α-KG) helps in supplying citrate for de novo lipogenesis. This pathway uses mitochondrial and cytosolic isoforms of isocitrate dehydrogenase	^35^
H	Liver, cervical, bronchial smooth muscle	Hypoxia caused TG accumulation by HIF-1-mediated stimulation of LIPIN1 expression	^43^
H	Prostate	Hypoxia-induced TG accumulation in extracellular vesicles (EVs) released from prostate cancer cells; upregulated expression of markers for FA synthesis (ACLY, FASN and ACACA) and FA desaturation (SCD-1); phospholipid and TG profiles both altered in cells and EVs; saturation index of membrane phospholipids increased	^28^
H	Cervical	Phosphatidylcholine profiles and the level of individual species were altered; relative abundance of phospholipid species with acyl chains containing ≥3 double bonds not significantly different from those containing <3 double bonds	^31^
H	Leukaemia, colon, lung	Cancer cells maintain lipid class homoeostasis under hypoxic stress. The levels of individual lipid moieties alter under hypoxia, but the robust averages of the broader lipid class remain unchanged	^69^
H	Ovarian	FABP4 expression was increased	^42^
H	Clear cell renal cell carcinoma	Carnitine palmitoyltransferase 1A expression is repressed, reducing FA transport into the mitochondria, and forcing FAs to accumulate lipid droplets for storage	^49^
LS	Breast	Cancer cells more dependent on de novo lipid synthesis	^12^
LS	Breast, prostate	De novo FA synthesis upregulated; cellular levels of MUFA increased	^60^
LS	Lung, pharynx, lung	SCD-1-mediated FA desaturation upregulated	^50^
LS	Leukaemia, colon, lung	In leukaemia cells neutral lipid compositions were markedly modified. Cellular level of TG subspecies decreased with increasing number of double bonds in their fatty acyl chains. Colon and lung cancer cells showed overall decrease in cholesterol ester under serum deprivation. A similar trend was observed under LS + H conditions	^69^
LS	Kidney	Significant reductions in TGs and cholesterol ester levels; decreases in the abundance of unsaturated TGs and a shift towards TG saturation	^70^
LL	Brain	Expression of SREBPs upregulated	^11^
LL	Prostate, lung, liver	Increased dependency on de novo FA synthesis for cell survival	^73^
LL	Breast, prostate	Expression of ACSS2 upregulated	^12^
LL	Pharynx, lung	Expression of SCD-1 upregulated	^50^
LL	Prostate, lung, liver, renal	Expression of markers for de novo FA synthesis (ACLY and FASN) and mevalonate synthesis (HMGCR) upregulated; expression of ACSS2 also upregulated	^13^
LL	Brain	Low effect on lipid droplet accumulation	^10^
LL	Haematopoietic	No effect on cellular cholesterol levels	^74^
LL	Haematopoietic	Cellular cholesterol levels unaffected; TG levels significantly elevated	^75^
MEM	Breast, prostate	Cancer cells primarily dependent on de novo FA synthesis; phosphatidylcholine and phosphatidylethanolamine profiles altered: the levels of phosphatidylcholines and phosphatidylethanolamines with shorter, more saturated fatty acyl chains increased	^12^
LS + H	Breast, prostate	Increased acetate-dependent FA synthesis	^12^
LL + H	Brain	Increased expression of markers for FA synthesis (FASN, ACACA, ACACB), desaturation (SCD-1) and uptake (FABP3 and 7); expression of HMGCR also upregulated	^11^
LS + H	Breast	Cancer cells utilised most of the acetate for synthesis of acetyl-CoA; ACSS2 mainly localised in the nucleus, where it recaptures acetate released from histone deacetylation for recycling by histone acetyl transferase	^65^
LL + H	Brain	Low effect on lipid droplet accumulation	^10^
LS + H	Kidney	Decrease in TGs harbouring unsaturated FAs and a shift towards increased TG saturation; saturation of diacyglycerols also increased	^70^

Reference numbers also correspond to the reference numbers in Fig. 1

*H* hypoxia, *LS* low serum, *LL* low lipid, *MEM* minimum essential medium

In hypoxic cancer cells, the expression of other enzymes that are involved in de novo lipid synthesis often show similar trends to those of FASN (Table 1). Most of these enzymes are under the transcriptional control of sterol regulatory-element binding protein-1 (SREBP-1). Hypoxia induces the activation of Akt and HIF-1, which is followed by the increased expression and activation of SREBP-1, which in turn induces the increased expression of FASN, among other biosynthetic enzymes.^26^ Conversely, the studies that reported a hypoxia-induced reduction in FASN expression showed that SREBP-1 expression is also decreased or unaffected under such conditions.^11,29^

In normoxic cells, glucose is the main source of acetyl-CoA for downstream lipid synthesis pathways (Fig. 1; FA synthesis and mevalonate pathway). However, hypoxia inhibits the entry of glucose-derived pyruvate into the TCA cycle,^19,33^ which consequently prevents glucose-based acetyl-CoA synthesis.^19^ To compensate, cancer cells adopt different metabolic mechanisms for FA synthesis—for instance, relying on glutamine or acetate as alternative substrates.^34–36^ Hypoxic breast, prostate, cervical, lung and colon cancer cells show increased acetate uptake, which has been shown to support tumour growth.^12,34,37^ Recent studies provide evidence that acetate is a fundamental nutrient that can fuel cancer growth under metabolic stress. Acetate can be endogenously generated through the removal of acetyl groups from histones by histone deacetylases^38^ and through hydrolysis of acetyl-CoA.^39^ It was recently shown that more than 80% of the intracellular acetate in cancer cells is generated de novo through glycolysis.^40^ While the expression of acyl-CoA synthetase short-chain family member 2 (ACSS2)—an enzyme that produces acetyl-CoA using acetate as a substrate—was found to be upregulated in hypoxic breast and prostate cancer cells,^12,37^ it was downregulated in hypoxic liver cancer cells;^41^ however, acetate supplementation increased ACSS2 and ACSS1 expression.^41^ These contrasting observations were attributed to the differences in cell culture methods. In addition, it has been shown that in hypoxic HepG2 cells acetate also functions as an epigenetic metabolite that enhances H3 acetylation levels at the promoter regions of FASN and acetyl-CoA carboxylase α (ACACA), resulting in increased expression of these genes, thus enhancing FA synthesis (Fig. 1; Nucleus).^41^ Interestingly, ACSS1 and ACSS2 were found to be involved in this acetate-induced epigenetic regulation of FA synthesis.^41^ Clinical samples of hepatocellular carcinoma with high ACSS1 and ACSS2 expression exhibit increased histone H3 acetylation and FASN expression.^41^ Thus, ACSS1 and ACSS2 were suggested to be potential therapeutic targets for cancer. It has been shown that co-silencing of FASN and ACSS2 induces cell death in biologically diverse cancer cell lines.^13^

### Exogenous FA uptake under hypoxic conditions

In addition to adjusting for different substrates, hypoxic cancer cells might also use FA uptake pathways to compensate for reduced glucose-based de novo FA synthesis.^3,9,10^ Hypoxia increases FA uptake in breast cancer, ovarian cancer and glioblastoma cells by inducing the expression of FA-binding proteins (FABP3, FABP7 or FABP4), which are involved in the uptake and subcellular trafficking of FAs^10,11,42^ (Fig. 1, Table 1). Hypoxic cancer cells are known to accumulate lipid droplets.^43^ Bensaad et al.^10^ reported that this hypoxic accumulation of lipid droplets in breast cancer and glioblastoma cells is mediated by FABP-dependent FA uptake, while de novo FA synthesis is repressed. Gharpure et al.^42^ demonstrated that microRNA-409-3p regulates the expression of FABP4 in ovarian cancer cells. Hypoxia reduces the expression of microRNA-409-3p, thus potentially removing its inhibitory effect on FABP4, which subsequently results in increased FABP4 levels. These FABPs have been shown to be involved in tumour progression.^10,42^ In murine models, inhibition of FABP3,^10 ^FABP7^10^ and FABP4^42^ impairs in vivo tumorigenesis.^10^ Clinical data also show that high FABP4 expression is significantly correlated with poor overall and progression-free survival.^42,44^

Expression of CD36, an FA uptake channel, is known to be upregulated under hypoxia in different non-transformed cells.^45,46^ CD36 has been previously implicated in tumour progression.^7,8,47^ Metastasis-initiating cells in human oral carcinomas display high levels of the CD36.^48^ Clinical data show that the presence of CD36+ metastasis-initiating cells correlates with a poor prognosis for numerous types of carcinomas, and inhibition of CD36 also impairs metastasis, at least in human melanoma- and breast cancer-derived tumours.^48^ Nevertheless, hypoxic regulation of CD36 in cancer cells is not studied in detail. Du et al.^49^ showed that at least in RCC4 clear cell renal cell carcinoma cells, hypoxic accumulation of lipid droplets was not mediated by CD36. Further studies are required to study this phenomenon in detail.

Hypoxia has been shown to increase FA uptake, mainly of monounsaturated FAs (MUFAs), in diverse cancer cell lines (Fig. 1).^9^ The reason for this specific MUFA uptake is not clearly understood, although it has been shown that cancer cells require MUFAs for survival.^50^ A specific balance between saturated FAs (SFAs) and unsaturated FAs is also critical for cancer cell progression. Alteration in the ratio of  SFAs to MUFAs in cellular FA pools affects cell survival and proliferation via a number of interrelated mechanisms, including perturbations in mitochondrial function, heightened cellular reactive oxygen species (ROS), ER stress and apoptosis.^51–53^ FAs synthesised de novo are initially fully saturated; hence, a substantial fraction of de novo synthesised FAs will require desaturation by the activity of stearoyl-CoA desaturase-1 (SCD-1), a process that notably requires oxygen. Hypoxia therefore renders cells dependent on the uptake of exogenous unsaturated FAs. Indeed, limiting the supply of exogenous FAs to hypoxic cells leads to a critical deficiency in unsaturated FAs and results in cell death induced by endoplasmic reticulum stress.^54^ Thus, inhibiting FA uptake in hypoxic areas within tumours could represent a promising target for anticancer therapy. Indeed, a recent study has identified tamoxifen as a potential drug of interest that could inhibit FABP4 and subsequently affect migration of ovarian cancer cells.^42^

### Lipid droplets and hypoxia

FAs acquired by cancer cells, either by endogenous synthesis or by exogenous uptake, are rapidly incorporated into cellular triglycerides (TGs), which form the core of lipid droplets in cells. Hypoxia-mediated accumulation of TGs and lipid droplets is accompanied by the increased expression of LIPIN1,^43^ an enzyme that catalyses the conversion of phosphatidic acid into diacylglycerol in the penultimate step of TG synthesis (Fig. 1; TG Synthesis). Hypoxia also promotes the storage of lipids in lipid droplets through the induction of perilipin 2 (PLIN2).^10^

Upon demand, fatty acyl moieties can be released from these stored TG deposits in lipid droplets via an intracellular lipolytic pathway (Fig. 1; Intracellular Lipolysis).^55^ Zechner et al.^55^ showed that monoglyceride lipase (MGLL) provides, by de-esterification, a stream of intracellular free FAs that can fuel cancer cell proliferation. Zhang et al.^32^ observed that intracellular lipolysis mediated by patatin-like phospholipase domain containing 2 (PNPLA2) [commonly known as adipose triglyceride lipase (ATGL)] is significantly reduced under hypoxic conditions.^32^ This lipolytic inhibition contributes to the accumulation of TGs and lipid droplets and cancer cell survival. The expression of PNPLA2 in hypoxic cancer cells remains unchanged; instead, PNPLA2 activity is inhibited by the protein encoded by hypoxia-inducible gene 2 (HIG2), a HIF-1 target.^32^ Experiments with murine xenograft models demonstrated that this inhibition of lipolysis by HIG2 is critical for tumour growth in vivo.^32^ Moreover, an abundance of HIG2 mRNA was observed in clinical samples collected from patients with renal clear cell carcinoma, colorectal adenocarcinoma, lung squamous cell carcinoma, bladder urothelial carcinoma, and uterine corpus endometrial carcinoma,^32^ indicating that PNPLA2 inhibition by HIG2 is a relevant mechanism for cancer pathophysiology in humans. As the accumulation of excess FAs can induce lipotoxicity, which might be especially important during the periods of hypoxia when FA uptake is upregulated, storage of excess FAs in lipid droplets through inhibition of PNPLA2-mediated lipolysis would constitute a conceivable strategy for cancer cells to evade lipotoxicity during hypoxic conditions.

### FA oxidation under hypoxia

PNPLA2 also activates the FA oxidation pathway, leading to energy production, in normoxic cells.^55^ The de novo synthesis of FAs is considered an essential pathway for cancer cell survival and, according to the classical view, FA synthesis and FA oxidation cannot occur together. More recently, however, studies have challenged this notion and suggest that both metabolic pathways can be active simultaneously and independently of each other (reviewed in ref. ^56^). Under normal conditions, PNPLA2 channels excess FAs into the FA oxidation pathway by inducing the expression and activity of peroxisome proliferator-activated receptor-α (PPAR-α), a transcription factor and major regulator of lipid metabolism. However, sustained FA oxidation often results in increased generation of ROS, which may lead to oxidative stress and cell death.^32^ To counteract the damaging effects of ROS, PPARα also promotes the expression of various anti-oxidases, such as catalase and superoxide dismutase.^57,58^ In normoxia, mechanisms balancing ROS generation and degradation may maintain a steady-state redox environment. However, during hypoxia, a tilt towards excessive ROS production can occur as a result of increased electron leakage from the mitochondrial electron transport chain, leading to oxidative stress. Hence, inhibition of PNPLA2 activity by HIG2 under conditions of hypoxia also promotes cancer cell survival by reducing FA oxidation, ROS overproduction and oxidative damage. HIG2 is proposed to be a novel metabolic oncogenic factor that exerts its function by neutralizing the tumour suppressive role of PNPLA2. Development of drugs that disrupt HIG2-PNPLA2 interaction would liberate PNPLA2 and potentiate FA oxidation-driven ROS production to toxic levels, resulting in apoptotic death of hypoxic cancer cells.

A recent study^49^ showed that hypoxia also represses the expression of carnitine palmitoyltransferase 1A—a rate-limiting enzyme in long-chain FA oxidation—in clear-cell renal cell carcinoma. This reduces FA transport into the mitochondria and forces FA transport into lipid droplets for storage.

Huang et al.^27^ have reported another mechanism suppressing FA oxidation in hypoxia, by HIF-1α-mediated inhibition of acyl-CoA dehydrogenase medium-chain (ACADM) and acyl-CoA dehydrogenase long-chain (ACADL) expression. ACADM and ACADL catalyse the first step of FA oxidation in mitochondria (Fig. 1; β-Oxidation) but differ in the chain length of their FA substrates. Furthermore, ACADL is known to mediate unsaturated FA oxidation, whereas ACDM prefers SFAs as substrates. Interestingly, depletion of ACADL, but not ACADM, by HIF-1α promotes the progression of cancer cells, potentially by mediating the accumulation of unsaturated FAs. This observation was also found to be relevant in clinical settings. ACADL expression is downregulated in clinical hepatocellular carcinoma (HCC) samples in comparison to normal adjacent tissues.^27^ ACADL expression was shown to further decrease as HCC progressed to a higher clinical stage. ACADL expression in HCC patients was also linked to survival time, with patients expressing high levels of ACADL in their HCC lesions surviving much longer than those with low expression levels.

### Extracellular lipolysis

Different types of cancer cells also express lipoprotein lipase (LPL), a lipolytic enzyme that is involved in the extracellular lipolysis of TG-rich lipoproteins (TGRL). LPL—bound to a heparin-like heparan sulfate proteoglycan motif either on the cancer cell surface or on the luminal surface of the vascular epithelium—has been speculated to release FAs from TGRLs (Fig. 1; Extracellular Lipolysis).^7,8^ These FAs can then enter the cancer cells via CD36, an FA uptake channel. In normal human preadipocytes, acute hypoxia strongly inhibits LPL-mediated lipolysis.^59^ LPL-regulated extracellular lipolysis in cancer cells may facilitate the hypoxia-induced FA uptake by rapidly releasing FAs from TGRLs in the tumour microenvironment. However, hypoxia-mediated regulation of LPL-regulated extracellular lipolysis in cancer cells has not been investigated in detail.

### Lipid metabolism under conditions of nutrient deprivation

Poor blood perfusion in tumours reduces the availability of serum-derived factors and nutrients to the cancer cells, which might affect their lipid metabolism pathways. Regulation of the FA synthesis pathway in cancer cells cultivated in low-serum conditions has been studied (Table 1). Under such conditions, the availability of FAs, among several other nutrients, is restricted, making the cancer cells more reliant on endogenous synthesis for FA acquisition.^12,60^ As discussed above, the proliferation of various cancer cell types requires SCD1-mediated desaturation of endogenously synthesised SFAs into MUFAs, and SCD1, in turn, requires oxygen, so that, under oxygen-deprived conditions, cancer cells rely on an exogenous supply of MUFAs. However, in low-serum conditions, in which the exogenous supply of MUFAs is restricted, cancer cells instead upregulate their expression of SCD1 to facilitate increased endogenous FA desaturation. It has been reported that SCD1 inhibition induces cytotoxic effects on cancer cells in low-serum conditions, while it has little impact on cells cultured in full-serum conditions.^50,60^

This increased dependence of cancer cells on de novo FA synthesis and desaturation in low-serum conditions is thought to be mainly caused by the decreased availability of lipids/FAs. To further explore this hypothesis, we specifically investigated the effects of lipid deprivation on FA synthesis in cancer cells (Table 1) by cultivating cancer cells in media depleted of lipids or lipoproteins. We observed that in such conditions cancer cells differentially activated and thrived on endogenous lipid synthesis pathways.^13^ As expected, the expression and activity of SCD1 were also elevated.^50^ Therapeutic inhibition of SCD1 has been shown to efficiently reduce cancer cell proliferation by selectively depleting MUFAs.^60–62^ The inhibition of SCD1 increases the susceptibility of cancer cells towards chemotherapeutic drugs and metabolic inhibitors. Interestingly, it was shown that SCD inhibition is only detrimental to prostate cancer cell survival (in vitro) in the absence of exogenous lipids, particularly oleic acid.^60^ However, silencing of SCD reduces tumour growth in xenograft models of lung,^63^ gastric^50^ and liver cancer^64^, and also inhibits orthotopic growth of prostate cancer cells in vivo.^60^ Taken together, these studies suggest that unsaturated lipids are indeed limited within the tumour microenvironment. Further studies are required to demonstrate the effects of serum and lipid deprivation on other pathways involved in lipid metabolism.

### Synergistic effects of hypoxia and nutrient deprivation on lipid metabolism

The combinatorial effects of hypoxia and nutrient deprivation, conditions that more closely mimic the in vivo tumour microenvironment, on de novo FA synthesis in cancer cells have been studied (Table 1). In such conditions, cancer cells upregulate de novo FA synthesis.^11,12^ As hypoxia also prevents glucose-based acetyl-CoA generation, cancer cells depend on glutamine or acetate as an alternative substrate for acetyl-CoA generation in conditions of hypoxia combined with low serum growth, to fuel their elevated FA synthesis pathway.^12,35,65^ Indeed, FA synthesis was shown to be decreased under hypoxic conditions, but increased when cancer cells were cultivated under hypoxia in combination with serum deprivation.^11^ As expected, expression of ACSS2 is elevated under conditions of oxygen deprivation and serum deprivation, but is mainly localised in the nucleus (Fig. 1; Acetate Metabolism).^65^ ACSS2 serves a dual function when oxygen and serum are limited: it facilitates the consumption of extracellular acetate as an alternative carbon source, but the increased nuclear localisation also enables cells to retain much of their endogenously produced acetate, which in turn allows cells to maintain sufficient acetylation of histones, to prevent initiation of apoptosis, and to maintain growth.^65^ The specific role of ACSS2 in cells that are hypoxic or metabolically stressed opens a therapeutic window in which ACSS2 could be rendered specifically toxic only to tumours. The therapeutic feasibility of pharmacologically targeting ACSS2 is currently being explored.

### Effects of hypoxia and nutrient deprivation on lipid load and lipidomic profiles in cancer cells

As discussed above, hypoxic cancer cells modify the balance between FA synthesis and uptake, which leads to a significant accumulation of TGs and lipid droplets.^43^ Upon re-oxygenation, cells then use the lipids stored in droplets for energy production and antioxidant defence.^10^ However, the functional significance of the increased accumulation of lipid droplets during continued hypoxia is contentious. Hypoxic stress also promotes glycogen synthesis as a mechanism of storing glucose, while it is still available for later use as an anaerobic source of energy. However, as lipids can only produce energy through oxidative phosphorylation, a process drastically inhibited by hypoxia, a similar scenario may not arise in the case of lipids. Mylonis et al.^43^ proposed that TG storage by hypoxic cancer cells might help the cells to buffer the lipotoxicity caused by free FAs, which arises because of the suppressed respiratory-chain activity.^66,67^

Mammalian cells have a limited ability to synthesise polyunsaturated fatty acids (PUFAs) de novo, as they lack the Δ12 desaturase enzyme, and so cell membranes of lipogenic tumour tissues, with highly active de novo FA synthesis, are enriched in SFAs or MUFAs.^68^ As these FAs are less prone to lipid peroxidation than PUFAs, they make cancer cells more resistant to oxidative-stress-induced cell death^68^ and even to targeted therapies, including clinically used BRAF inhibitors. Furthermore, SFAs are packed more densely, and their increased levels are shown to alter membrane dynamics and limit drug uptake.^68^ Depletion of SFAs and MUFAs—by inhibiting FA synthesis—affects lateral and transversal membrane dynamics in cancer cells. It was shown that depletion of SFAs induces six-fold increase in the flip-flop rate of doxorubicin, accompanied by a significant increase in the intracellular accumulation of doxorubicin in prostate cancer cells.^68^ These findings suggest that tumour-associated FA synthesis protects the cancer cells against chemotherapeutic insults. They also highlight the significance of FA synthesis inhibitors as antineoplastic agents and chemotherapeutic sensitizers. Although several groups have studied the differential regulation of FA metabolism under metabolic stress, only a few have investigated lipidomic profiles^10,28,30,31^ (Table 1). It was reported that, under hypoxia and low serum conditions, the phosphatidylcholine and phosphatidylethanolamine profiles of breast cancer cells are altered, with an increase in the number of shorter and more saturated fatty acyl chains,^12^ indicating enhanced FA synthesis. HeLa cells, on the other hand, showed decreased levels of mono- and di-unsaturated, but increased levels of polyunsaturated, phospholipids (PLs).^31^

Schlaepfer et al.^28^ reported that hypoxic prostate cancer cells, and extracellular vesicles released by these cells, are significantly enriched in TGs due to the activation of the FA synthesis pathway. In addition, the TG sub-species profiles of these cells and the released extracellular vesicles were significantly altered. Cellular levels of myristic, palmitic and stearic FAs  (all SFAs) were increased. The levels of linoleic acid and its derivative arachidonic acid, essential FAs implicated in tumour progression, were also significantly increased in the TGs of hypoxic cancer cells. Moreover, the levels of palmitic, stearic, linoleic and arachidonic acid were elevated in extracellular vesicles compared to those under normoxic conditions. Upon reoxygenation these cells break down the intracellularly stored lipids, and the generated energy induces increase in cell proliferation and the invasiveness potential of these cells. Interestingly, this increased proliferation and invasiveness were compromised by blocking the arachidonic acid pathway.^28^ Another study reported that, under hypoxic conditions, cellular levels of TGs with three double bonds significantly decreased in MCF7 breast cancer cells,^10^ but significantly increased in U87 glioblastoma cells.^10^ A recent study^69^ indicates that lipidomic profiles of leukaemia cells are predominantly affected by serum deprivation. Neutral lipid compositions are markedly modified under serum deprivation and, strikingly, the cellular levels of TG subspecies decreased with increasing number of double bonds in their fatty acyl chains. In contrast, cancer cells maintained lipid class homoeostasis under hypoxic stress. It was shown that, although the levels of individual lipid moieties alter under hypoxia, the robust averages of the broader lipid class remain unchanged. Another recent study^70^ also reported that low serum level affects TG composition in renal cancer cells, with significant decrease in the abundance of unsaturated TGs and a shift towards TG saturation. The authors also reported that hypoxia in combination with low serum induces decrease in levels of TGs harbouring unsaturated FAs  and a shift towards increased TG saturation.

Hypoxia induces changes in the saturation index of membrane lipids in cancer cells that could affect cell membrane fluidity, dynamics and drug resistance. The role of hypoxia in drug resistance has been well known for at least 60 years now.^71,72^ Lipidomic analyses may also provide an explanation for the changes in membrane fluidity and dynamics observed in hypoxic cancer cells. The significance of alterations in TG subspecies profile in the context of tumour biology is being evaluated. Further studies, using broader lipidomic assays and larger panels of cell lines, are required to get a holistic view of lipidomic profiles in cancer cells under metabolic stress. These studies will not only help in understanding the role of lipids in cancer progression but also promote clinical applications of lipidomic profiling, including identification of potential therapeutic targets and diagnostic markers.

## Conclusions

**Fig. 2:**
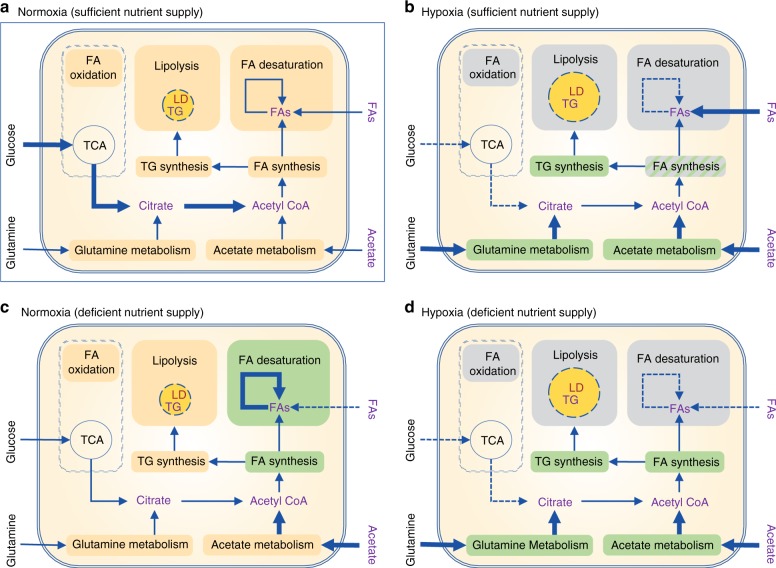
Effect of oxygen deprivation and/or nutrient deprivation on fatty acid (FA) metabolism in cancer cells. **a** Cancer cells with a sufficient supply of nutrients and oxygen mainly use glucose-derived acetyl-CoA for de novo FA synthesis to support rapid cell proliferation. They can also acquire FAs from the environment. **b** Different types of hypoxic cancer cells differentially regulate FA synthesis depending on various environmental factors—particularly, nutrient availability. Hypoxia inhibits the entry of glucose-derived pyruvate into the TCA cycle. Hence, cells either switch to alternative carbon sources (i.e. glutamine or acetate) for FA synthesis or increase their FA uptake. FA desaturation is impaired by oxygen deprivation; therefore, the uptake of unsaturated FAs is particularly enhanced. FAs are rapidly incorporated into cellular triglycerides (TGs) that enhance TG and lipid droplet (LD) accumulation. **c** Under nutrient and lipid restriction cancer cells mainly rely on endogenous FAs and desaturation. **d** When hypoxia is induced in combination with nutrient and lipid deprivation, cells cannot acquire FAs from the environment. Hence, they switch back to de novo FA synthesis, but fully depend on glutamine or acetate as an alternative substrate. Parts (**b**), (**c**) and (**d**) are drawn in comparison to the normoxic state depicted in (**a**). The line thickness represents the level of flux through the pathway. Dashed lines indicate biosynthetic pathways that are inactive due to lack of substrates or cofactors. The colours of the pathway boxes in (**b**), (**c**) and (**d**) represent upregulation (green), downregulation (grey) or differential regulation (green/grey) of the corresponding pathway in comparison to the normoxic state (with sufficient nutrient supply) in (**a**)

Dysregulation of lipid metabolism pathways in cancer cells has been widely reported. Multiple studies have indicated that such alterations are modulated by various cancer cell-intrinsic processes. However, emerging evidence suggests that these modifications are also mediated by crosstalk between the tumour microenvironment and metabolic circuits within cancer cells. The tumour microenvironment is mostly hypoxic. Although a few studies indicate that cancer cells might increase FA synthesis under hypoxia, most recent studies suggest that hypoxic cancer cells switch from endogenous FA synthesis to increased exogenous FA uptake because of the inhibition in glucose-based acetyl-CoA generation (Fig. 2). Alternatively, certain cancer cells, to compensate for this downregulation of glucose-based acetyl-CoA synthesis, switch to other carbon sources, such as glutamine or acetate. Nevertheless, FA desaturation by SCD1 requires oxygen and is therefore inhibited under hypoxic conditions, rendering cancer cells dependent on exogenous unsaturated FAs. In addition to the increased uptake of MUFAs, intracellular lipolysis and FA oxidation are downregulated under hypoxia, which results in the increased accumulation of TGs in lipid droplets. Increased TG synthesis and decreased lipolysis are both suggested to be strategies adopted by different cancer cells to evade lipotoxicity during hypoxia.

Nutrient and lipid deprivation renders cancer cells completely dependent on endogenous FA synthesis and desaturation. However, in conditions of hypoxia combined with nutrient and lipid deprivation, exogenous lipids are also in short supply. Hence, the cells switch back to de novo FA synthesis, but fully depend on glutamine or acetate as an alternative substrate. Different cancer cells may adopt different mechanisms to thrive under metabolic stress. Nevertheless, therapeutic targeting of de novo FA synthesis in cancer cells would be most effective under conditions that limit metabolic flexibility or, possibly, by concurrently targeting several routes of lipid acquisition. With the advent of better molecular techniques, including mass spectrometry imaging, to spatially visualise lipids in intact tissues, it will be possible to address several of the outstanding questions, to better correlate altered lipid profiles with changing microenvironments and to better explore the potential of lipid metabolism as an antineoplastic approach.

## Supplementary information

Supplementary Table

## Data Availability

Not applicable
