# Supplementary material for: Lipid metabolism in cancer cells under metabolic stress
Source: Br J Cancer. 2019 May 16;120(12):1090–8. doi: 10.1038/s41416-019-0451-4 (PMC6738079; doi:10.1038/s41416-019-0451-4)
Supplement: Supplementary file 1 — Supplementary Table [file 41416_2019_451_MOESM1_ESM.docx]

| **Supplementary Table 1: Effect of metabolic stress conditions on regulation of lipid metabolism in cancer cells.** | | | | | | | | | |
| --- | --- | --- | --- | --- | --- | --- | --- | --- | --- |
| **Stress** | **Stress Induction** | **Cancer Type** | **Cell lines** | **Effect on Lipid Metabolism** | **Effects on lipid/lipidomic profiles** | **Other Relevant Observations** | **Effects confirmed in 3D culture/xenograft models/clinical tissue samples** | **References** | **References in text** |
| **H** | GasPak induced hypoxia.  **Incubation time**  48 hours | Breast | MX1  MCF7  MDA-MB157 | - **↑FASN** (FA synthesis) - **↑** **SREBP-1**, **HIF1**(TFs) - **↑p-SREBP,** , **p-Akt** (TFs) |  | - **╣** **HIF1** (By YC-1) blocks the expression of FASN and SREBP. - **╣** **Akt** (By LY294002) blocks the expression of **FASN**, **SREBP**, **p-Akt** and **HIF1**. | **Mouse Xenograft model**   - In xenograft mouse model, FASN was strongly expressed in the hypoxic regions of the tumor.   **Clinical tissue samples**   - In human breast tumor specimens both FASN and SREBP-1 were colocalized with hypoxic regions in the tumors. | [^1^](#_ENREF_1) | 26 |
| **H** | **O_2_ Concentration**  1% O_2_  **Incubation time**  48 hours. | Liver  Prostate | Hep3B  SK-Hep-1  HepG2  PC3M | - **↓ ACADM** & **ACADL** (Beta Oxidation) - **↑FASN** - **↑LPIN1** (TG synthesis) - **↑** **SCD1** (FA desaturation) - **↓FABP7** (FA uptake) | - **↑**TGs levels | - **╨HIF-1α** and **HIF-2α** results in **↓** TGs levels in Hep3B cells. | **Mouse Xenograft model**   - **╨**LCAD enhanced the tumor growth. - **╨**MCAD **↔** tumor growth. - **╨** LCAD and MCAD induced lipid accumulation.   **Clinical tissue samples**   - **↓**LCAD in tumor tissues. - **↑**HIF-1α, Akt phosphorylation - **↓** PGC-1b, LCAD, PTEN protein levels in tumor tissues. - LCAD expression **∞**disease progression. | [^2^](#_ENREF_2) | 27 |
| **H** | **O_2_ Concentration**  2% O_2_  **Incubation time**  12 hours. | Liver | HepG2 | - **↓FASN** (only in high-density cell culture under cytotoxic stress) - **↔ FASN** (low-density cell culture) - **↓SREBP** |  |  |  | [^3^](#_ENREF_3) | 29 |
| **H** | **O_2_ Concentration**  <0.5% O_2_  **Incubation time**  24 hours. | Brain | U87  U251 | - **↓ACACA**, **ACACB**, **FASN** (FA synthesis) - **↓HMGCR** (Mevalonate synthesis) - **↑SREBPF2** - **↓SREBPF1c** - **↔SREBPF1a** - **↑** **SCD** (FA desaturation) - **↑FABP3**, **FABP7** (FA uptake) - **SCD** and **FABP3** expression was correlated with **┤** **SREBP1**. - **FABP7** expression was correlated with **┤** **SREBP1** & **SREBP2**. |  |  |  | [^4^](#_ENREF_4) | 11 |
| **H** | **O_2_ Concentration**  Hypoxia: 1% O_2_  **Incubation time**  24 hours. | Colorectal | HCT-116  SW1222  DLD1 | - **↓** **ACACA** (FA synthesis) - **↔FASN** - **↑ SCD-1** (FA desaturation) - **↑SREBP-1** | - **↑** FAs levels - **↓** Desaturation index |  |  | [^5^](#_ENREF_5) | 30 |
| **H** | **O_2_ Concentration**  Hypoxia: 1% O_2_  **Supplementations**  2.5mM acetate  **Incubation time**  12 hours. | Liver  Breast  Prostate | HepG2  A549  PLC-8024  SkBr3  DU 145 | - **↑ ACACA** - **↑FASN** - **↑ACSS2** (Acetate-dependent acetyl Co-A synthesis) - Acetate fuels *de novo* FA synthesis which promotes the cancer cell survival. - **↑** Acetate uptake**.** - Acetate epigenetically activates FA synthesis. |  | - **╨FASN** results in   **↓**Acetate derived *de novo* FA synthesis.   - HepG2/SkBr3 cells were more sensitive to FASN inhibitor C75 | **Clinical tissue samples**   - Higher % of liver cancer tissues displays ↑expression of **ACSS1** as compared to **ACSS2**. - **↑** **H3K9ac, H3K14ac, H3K27ac & H3K56ac** expression in ACSS2 expressing tumors. - H 3 acetylation and FASN expression is correlated withACSS1/2 expression. | [^6^](#_ENREF_6) | 41 |
| **H** | **O_2_ Concentration**  Hypoxia: 0.1% O_2_  **Incubation time**  48 hours. | Breast Brain | MCF7  MDA-MB-231  U87  T98G | - **↑FABP3** and **FABP7** (FA uptake) - **↑ PLIN2** (LD Synthesis) - FA synthesis was repressed. | - **↑**LDs - **↑**TGs - **↓** TGs species with 3 double bonds in MCF7 cells. - **↑** TGs species with 3 double bonds in U87 cells three - **↑** Palmitate, Stearate and Linoleate levels. | - **┤FABP3**, **FABP7**, and **PLIN2** results in **↓**LD - **┤HIF-1α** **↓**LD - Treatment of DMOG activates HIF-1α and restores the LD levels. | **3D cell culture**   - **↑ FABP3**, **FABP7** & **PLIN2** protein expression in hypoxic core of tumor spheroids. - **↑** LD levels in hypoxic core of tumor spheroids.   **Mouse Xenograft model**   - **╨**FABP3 and FABP7 inhibit the growth of tumors. - **╨**FABP3 and FABP7 inhibit LD accumulation in tumors. | [^7^](#_ENREF_7) | 10 |
| **H** | **O_2_ Concentration**  Hypoxia: 1% O_2_  **Incubation time**  72 hours. | Breast  Cervical  Lung | MDA-MB-468  HeLa  A549 | - Cancer cells utilized glutamine as chief carbon source for synthesis of Acetyl-Co A. - Increase in FA uptake (particularly of MUFA (C18:1) | - **↑** Exogenous FA levels. - **↑** MUFAs (C18;1) levels | - **↓SCD-1** activity. | **Mouse Xenograft model**   - **╣** SCD1(CAY10566) effects the growth of Akt driven tumors. | [^8^](#_ENREF_8) | 9 |
| **H** | **O_2_ Concentration**  Hypoxia: 0.5% O_2_  **Supplementations**  200 µM Oleate/0.2% BSA complex.  **Incubation time**  24 hours. | Renal  Colorectal | ACHN  Caki-1  DLD-1  HCT116 | - **↔ PNPLA2 (Lipolysis)** - **↔ ABHD5** (**TF)** - **↔ G0S2** (**TF)** - Intracellular lipolysis is suppressed due to inhibition of **PNPLA2** by HIG2. | - **↑**TG levels |  | **Mouse Xenograft model**   - **╨**HIG2 delayed tumor growth. - **↑**Neutral lipid in HIG 2 KO tumors. - **↔** Lipid in ATGL KO tumors.   **Clinical tissue samples**   - **↑** **HIG 2** in kidney, lung, colon, bladder and uterine tumor tissues. - **↑** **HIG 2** in RCC tissues as compared to adjacent normal kidney tissues. - **↔ PNPLA2** & **ABHD5** in RCC tissues. | [^9^](#_ENREF_9) | 32 |
| **H** | **O_2_ Concentration**  Hypoxia: 1% O_2_  Cells and media were placed in low oxygen overnight.  **Incubation time**  48 hours. | Breast  Cervical  Lung | MDA-MB-468  HeLa  A549 | Cancer cells utilized glutamine and acetate for the synthesis of Acetyl-Co A. |  |  |  | [^10^](#_ENREF_10) | 34 |
| **H** | **O_2_ Concentration**  Hypoxia: 1-3% O_2_  **Incubation time**  48 hours. | Lung  Breast  Skin  Colorectal | A549  H460  MDA231  SK-MEL-5  A431  HCT-116 | Reductive carboxylation of glutamine-derived α-Ketoglutarate (α-KG) is responsible for supplying citrate for *de novo* lipogenesis. |  |  |  | [^11^](#_ENREF_11) | 35 |
| **H** | **O_2_ Concentration**  Normoxia: 1 % O_2_  **Incubation time**  48 hours. | Liver  Cervical  Bronchial smooth muscle | Huh7  HeLaM  Hbsm | - **↑LIP1N1** - **HIF1-α** & **EPAS1** expression correlated with **LIP1N1** RNA expression. | - **↑** TGs levels that are correlated with **LIP1N1** RNA expression. | - **┤LIP1N1** decreases the TGs accumulation. |  | [^12^](#_ENREF_12) | 43 |
| **H** | **O_2_ Concentration**  Hypoxia: 1% O_2_  **Incubation time**  1-72 hours.  *For EVs isolation cells were cultured in exome-depleted FBS | Prostate | LNCaP  DU145 | - **↓**Cell proliferation - **↑ FASN** - **↑ACACA** - **↑ACLY** - **↑SCD-1**   **After Reoxygenation**   - **↑**Cell proliferation | **Cells**   - **↑** Saturation index of membrane PL. - **↔**TGs levels. - **↑**Myristic, Palmitic, stearic, linoleic and arachidonic acid. - **↓**Lipid accumulation (after reoxygenation)   **Extracellular vesicles**   - **↑** Stearic, Palmitic and linoleic acid. - **↑** TGs levels.   **Cell vs. EVs**   - **↑**% age of saturated FA in PL in EVs in comparison to parental cells. - **↓** Stearic acid and Oleic acid in TGs in EV in comparison to parental cells. |  |  | [^13^](#_ENREF_13) | 28 |
| **H** | Hypoxia was induced by adding 200µM cobalt chloride  **Incubation time**  3 hours  *Cells were serum starved before incubation under hypoxia. | Cervical | HeLa cells |  | - Principal component analysis reveal clear-cut separation between lipidomic profiles of hypoxic and normoxic cells. - PL profile altered but changes were only observed when individual lipid-moieties were compared. - **↑**PL with polyunsaturated acyl chains. - **↓**PL with mono/di-unsaturated acyl chains. |  |  | [^14^](#_ENREF_14) | 31 |
| **H** | **O_2_ Concentration**  Hypoxia: 2 % O_2_  **Incubation time**  48 hours. | Leukemia  Colon  Lung | KG1  KCL22  KU812  SW480  SW620  A549 | - **↓**Cell proliferation of SW480 and A549 cells - **↓ FASN** (KCL22 , KU812, SW620 & A549 cells) - **↔ FASN** (KG1 & SW480 cells) - **↓ HMGCR** (KU812, SW620 & A549 cells) - **↔ HMGCR** (KCL22, KG1 & SW480 cells)   **↔ MGLL**  **↔ LPL**  **↔ CD36** | - **↔**Lipidomic profiles of cancer cells |  |  | [^15^](#_ENREF_15) | 69 |
| **H** | **O_2_ Concentration**  Hypoxia: 1% O_2_  **Incubation time**  24-48 hours. | Ovarian | HeyA8 MDR | - **↑** **FABP4** |  |  |  | [^16^](#_ENREF_16) | 42 |
| **H** | **O_2_ Concentration**  Hypoxia: 1% O_2_  **Incubation time**  48 hours. | Clear cell renal cell carcinoma | RCC4 | - **↓ CPT1A** |  |  |  | [^17^](#_ENREF_17) | 49 |
| **LS** | **Serum Concentration**  1% FCS  **Incubation time**  24 hours | Breast | BT20  BT549  MDA-MB-231  MDA-MB-468  MCF7  T47D  SKBr3  MCF10 A | Cancer cells are more dependent on *de novo* lipid synthesis |  | - Cancer cells displayed increased sensitivity to **FASN** inhibitors (C75 & AZ22) in terms of proliferation rates. | **3D cell culture**   - **╣**FASN (by AZ22) was more effective against MCTS growth. | [^18^](#_ENREF_18) | 12 |
| **LS** | **Serum Concentration**  1% FCS  **Incubation time**  72 hours | Breast  Prostate | MDA-MB-468  MDA-MB-231  BT549  BT20  SKBr3  T47D  MCF7  MCF10 A  LNCAP  PC3  DU145  RWPE1 | - **↑** *De novo* fatty acid synthesis | - Membrane phosphoglycerides are mainly composed of mono-unsaturated acyl chains. - **↑MUFA** levels in DU145 cells. | - **┤SCD** results in **↓**cell number. - **┤SCD** results in **↑**MUFA & PUFAs levels and **↓**SFAs levels. However, oleic acid supplementation restores the relative proportions of FA classes. - **┤SCD** reduces the mono-unsaturated acyl chains in PE, PI and PS. Supplementation of oleic acid restores the concentrations of mono-unsaturated species in cancer cells. | **3D cell culture**   - **┤**SCD results in **↓**spheroid size. - **┤**SCD results in **↓** MUFAs & PUFAs in MCTs of DU145 cells.   **2D Vs. 3D(10%FCS )**   - **↑**poly-unsaturated DG in MCTs of T47D cells - **↑** Mono-unsaturated PI and LPA.   **Mouse xenograft model**   - ╨SCD causes **↓** growth of DU145 tumors. - ↑Unsaturated DG and PC species in BT474 tumors.   **Clinical tissue samples**   - **↑** SCD in breast and prostate tumor tissues. | [^19^](#_ENREF_19) | 60 |
| **LS** | **Serum Concentration**  2% FCS  **Incubation time**  48 hours | Lung  Pharynx  Lung | A549  FaDu  H1299 | - **↑ SCD-1** - **↔SCD-5** |  | - **┤SCD-1** induced apoptosis - **┤SCD-5** inhibition did not induce apoptosis. - **╣SCD-1** (with A939572) reduced the Proliferation of cells. Supplementation of media with oleic acid reversed the effect of A939572. | **Clinical tissue samples**   - **↑** SCD -1 in cancer tissues. | [^20^](#_ENREF_20) | 50 |
| **LS** | **Serum Concentration**  2% FBS  **Incubation time**  48 hours | Leukemia  Colon  Lung | KG1  KCL22  KU812  SW480  SW620  A549 | - **↓**Cell proliferation of all cell lines except SW620 - **↓ FASN** (A549 cells) - **↔ FASN** (KCL22 , KU812, KG1, SW480 & SW620 cells) - **↓ HMGCR** (A549 cells) - **↔ HMGCR** (KCL22 , KU812, KG1, SW480 & SW620 cells) - **↑ MGLL** ( SW480 & SW620 cells) - **↓ MGLL**(KG1 & KU812 cells) - **↔ MGLL**(KCL22 & A549cells) - **↑ LPL**(KU812 cells) - **↔ LPL**(SW480) - **↑ CD36**(KU812 cells) | - **↓ CE** levels - **↓ TGs subspecies** levels (Leukemia cell lines) - **↑DGs** levels(Leukemia cell lines) - **↑Highly saturated PCPs** levels(Leukemia cell lines) |  |  | [^15^](#_ENREF_15) | 69 |
| **LS** | **Serum Concentration**  0.5% FBS  **Incubation time**  72 hours | Kidney | A498 |  | - **↓** Overall TGs pool - **↓** unsaturated TGs - **↓** CEs - **↑** Saturation index of TGs | - **╣SCD-1** increased the TGs and DGs saturation. |  | [^21^](#_ENREF_21) | 70 |
| **LL** | **Serum Concentration**  1% LPDS  **Incubation time**  24 hours | Brain | U87  U251 | - **↑** **SREBF1a**, **SREBF1c** , **SREBF2** |  |  | **3D cell culture**   - **┤**SREBP1 results in **↓**spheroid diameter. | [^4^](#_ENREF_4) | 11 |
| **LL** | **Serum Concentration**  Lipid-reduced FBS  **Incubation time**  48-72 hours | Prostate  Lung  Liver | PC3M  HOP62  HepG2 | - Decreased proliferation rates - Increased dependency on DNL. |  | - **┤ACLY** induced apoptosis. |  | [^22^](#_ENREF_22) | 73 |
| **LL** | **Serum Concentration**  1% LPDS  **Incubation time**  72 hours | Breast  Prostate | BT474  DU145 | **↑ACSS2** |  |  | **Clinical tissue samples**   - **↑** ACSS2 expression in IDCs and ILCs tissues. - ACCS2 expression correlated with disease progression/ cancer stage in breast cancer patients. - **↑** ACSS2 expression in metastatic prostrate tumors as compared to primary tumors. - **↑** ACSS2 expression in tumors as compared to normal adjacent tissues. | [^18^](#_ENREF_18) | 12 |
| **LL** | **Serum Concentration**  2% CS-FBS  **Incubation time**  8 hours | Pharynx  Lung | FADU  H1299 | **↑** **SCD-1** |  | - Cells were more sensitive to inhibition of SCD-1 by A939572. | **Mouse Xenograft model**   - **╣**SCD-1(A939572) inhibits the tumor growth. | [^20^](#_ENREF_20) | 50 |
| **LL** | **Serum Concentration**  10% lipid-reduced serum  **Incubation time**  72 hours | Prostate  Lung  Liver Renal | PC3M  HOP62  HepG2  T24 | - **↑** **ACLY** - **↑** **FASN** - **↑** **ACSS2** - **↑** **HMGCR** - Only the cell lines that significantly elevated lipogenic activity maintained their proliferation rates. |  | - **↑** Sensitivity to Soraphen A and Simvastatin. | - **3D cell culture**   **↓**Growth of tumor spheroids. | [^23^](#_ENREF_23) |  |
| **LL** | **Serum Concentration**  1% LPDS  **Incubation time**  48 hours | Brain | U87 |  | - × LD levels | - **╣**HMGCR (by Fatostatin) results in **↓**LD accumulation. |  | [^7^](#_ENREF_7) | 10 |
| **LL** | **Serum Concentration**  10% LPDS  **Incubation time**  24 hours | Hematopoietic | CCRF-CEM  KG-1  MOLT-3  THP-1 |  | - **↔**Cholesterol levels |  |  | [^24^](#_ENREF_24) | 74 |
| **LL** | **Serum Concentration**  10% LPDS  **Incubation time**  24 hours | Hematopoietic | THP-1  PBMCs |  | - **↔**Cholesterol levels - **↑**TG levels |  |  | [^25^](#_ENREF_25) | 75 |
| **ND+H** | **O_2_ Concentration**  Hypoxia: 0.1% O_2_  Normoxia: 21% O_2_  **Serum/Supplementations**  SMEM media.  **Incubation time**  72 hours | Breast  Prostate | MDA-MB-468  DU145 | - Cancer cells primarily depend upon the *de novo* fatty acid synthesis. - **↓**Glucose utilization by TCA cycle. | - **↑**PC and PE with shorter more saturated FA acyl chains. |  |  | [^18^](#_ENREF_18) | 12 |
| **LS+H** | **O_2_ Concentration**  Hypoxia: 0.1% O_2_  Normoxia: 21% O_2_  **Serum Concentration**  1% FCS  **Incubation time**  72 hours | Breast  Prostate | BT474  BT474c1  DU145 | - Cancer cells used acetate as chief carbon source. - **↑ ACSS2** |  | Cancer cells more sensitive to **┤ACSS2**. |  | [^18^](#_ENREF_18) | 12 |
| **LL+H** | **O_2_ Concentration**  Normoxia:20% O_2_  Hypoxia:<0.5% O_2_  **Serum Concentration**  1% LPDS  **Incubation time**  24 hours | Brain | U87  U251 | - **↑** **ACACA**, **ACACB**, **FASN**, - **↑HMGCR** - **↑** **SREBF1a**, **SREBF1c** , **SREBF2** - **↑↑** **SCD** - **↑↑FABP3**, **FABP7** - **┤ SREBP1** increased the apoptosis of cancer cells. |  |  |  | [^4^](#_ENREF_4) | 11 |
| **LS+H** | **O_2_ Concentration**  Normoxia:21% O_2_, Hypoxia:1% O_2_  **Serum Concentration**  1% DFBS  **Incubation time**  48 to 72 hours  Medium contain 90 µMU ^13^C-acetate | Breast | MDA-MB-468  BT-474 | - Cancer cells utilized most of the acetate for synthesis of Acetyl-Co A. - Increase in nuclear localization of ACSS2. Nuclear ACSS2 recaptures acetate released from histone deacetylation for recycling by histone acetyl transferase. |  |  | **Mouse Xenograft model**   - In hypoxic regions of tumor tissues ACSS2 is prominently expressed in the nuclei of tumor cells. | [^26^](#_ENREF_26) | 65 |
| **LL+H** | **O_2_ Concentration**  Normoxia:21% O_2_, Hypoxia:0.1% O_2_  **Serum Concentration**  1% LPDS  **Incubation time**  48 hours | Brain | U87 |  | - × LD levels | - **╣**HMGCR (by Fatostatin) causes **↔**LD accumulation. |  | [^7^](#_ENREF_7) | 10 |
| **LS+H** | **O_2_ Concentration**  Normoxia:21% O_2_, Hypoxia:0.5% O_2_  **Serum Concentration**  0.5% FBS  **Incubation time**  72 hours | Kidney | A498 | - Cancer cells were more sensitive to **┤DGAT**( in terms of proliferation ) | - **↑**FA saturation index of TGs and DGs. | - **┤DGAT** leads to **↓**TGs and **↑**≥2 SFA TGs levels |  | [^21^](#_ENREF_21) | 70 |

**Key: Abbreviations: ACACA**, acetyl-CoA carboxylase 1; **ACACB,** acetyl-CoA carboxylase 2; **ACSS2,** acyl Co-A synthetase-2; **ADFP,** adipose differentiation protein; **ATGL,** adipose triglyceride lipase; **Cho,** choline; **CPT1A,** Carnitine palmitoyltransferase 1A; **CS-FBS,** charcoal striped fetal bovine serum; **DFBS ,** dialyzed fetal bovine serum; **DMOG,** dimethyloxalylglycine; **DNL,** de novo lipid synthesis; **FA,** fatty acids; **FABP3,** fatty acid binding protein 3; **FABP4,** fatty acid binding protein 4; **FABP7,** fatty acid binding protein 7; **FFA,** free fatty acids; **FASN,** fatty acid synthase; **FBS,** fetal bovine serum; **FCS,** fetal calf serum; **H,** hypoxia; **HIF-1α,** hypoxia-inducible factor 1-alpha; **HIF-2α,** hypoxia-inducible factor 2α; **HIG2,** hypoxia-inducible gene 2 protein; **HMGCR,** 3-hydroxy-3-methylglutaryl-CoA reductase; **LD,** lipid droplet; **LCAD,** long-Chain Specific acyl-CoA dehydrogenase; **LL,** low-lipid; **LS,** low-serum; **MAG,** monoacylglycerol ; **MCAD,** Medium-chain acyl-CoA dehydrogenase; **MCTs,** multicellular tumor spheroids; **MUFA,** monounsaturated fatty acids; **PBMCs,** peripheral blood mononuclear cells; **PC,** Phosphatidylcholines; **PE,** phosphatidylethanolamines; **Pcho,** propargyl-choline; **PI,** phosphatidylinositol; **PS,** phosphatidylserine; **PUFA,** polyunsaturated fatty acids; **RCC,** renal cell carcinoma; **SCD,** stearoyl-CoA Desaturase; **SFA,** saturated fatty acids; **SMEM,** spinner minimum essential medium; **SREBP,** sterol regulatory element-binding proteins; **TG,** triglycerides

**Symbols:** ↓, down regulated; ↑, up regulated; ↑↑, strongly up regulated; ↔, no effect; ×, low effect; ┤, gene knock down by siRNA; ╨, gene knock out; ╣, pharmacological gene inhibition

1. Furuta E, Pai SK, Zhan R, Bandyopadhyay S, Watabe M, Mo Y-Y *et al.* Fatty acid synthase gene is up-regulated by hypoxia via activation of Akt and sterol regulatory element binding protein-1. *Cancer research* 2008; **68**(4): 1003-1011.

2. Huang D, Li T, Li X, Zhang L, Sun L, He X *et al.* HIF-1-mediated suppression of acyl-CoA dehydrogenases and fatty acid oxidation is critical for cancer progression. *Cell reports* 2014; **8**(6): 1930-1942.

3. Jung SY, Jeon HK, Choi JS, Kim YJ. Reduced expression of FASN through SREBP‐1 down‐regulation is responsible for hypoxic cell death in HepG2 cells. *Journal of cellular biochemistry* 2012; **113**(12): 3730-3739.

4. Lewis C, Brault C, Peck B, Bensaad K, Griffiths B, Mitter R *et al.* SREBP maintains lipid biosynthesis and viability of cancer cells under lipid-and oxygen-deprived conditions and defines a gene signature associated with poor survival in glioblastoma multiforme. *Oncogene* 2015; **34**(40): 5128-5140.

5. Valli A, Rodriguez M, Moutsianas L, Fischer R, Fedele V, Huang H-L *et al.* Hypoxia induces a lipogenic cancer cell phenotype via HIF1α-dependent and-independent pathways. *Oncotarget* 2015; **6**(4): 1920.

6. Gao X, Lin S-H, Ren F, Li J-T, Chen J-J, Yao C-B *et al.* Acetate functions as an epigenetic metabolite to promote lipid synthesis under hypoxia. *Nature communications* 2016; **7**: 11960.

7. Bensaad K, Favaro E, Lewis CA, Peck B, Lord S, Collins JM *et al.* Fatty acid uptake and lipid storage induced by HIF-1α contribute to cell growth and survival after hypoxia-reoxygenation. *Cell reports* 2014; **9**(1): 349-365.

8. Kamphorst JJ, Cross JR, Fan J, de Stanchina E, Mathew R, White EP *et al.* Hypoxic and Ras-transformed cells support growth by scavenging unsaturated fatty acids from lysophospholipids. *Proceedings of the National Academy of Sciences* 2013; **110**(22): 8882-8887.

9. Zhang X, Saarinen AM, Hitosugi T, Wang Z, Wang L, Ho TH *et al.* Inhibition of intracellular lipolysis promotes human cancer cell adaptation to hypoxia. *eLife* 2017; **6**.

10. Kamphorst JJ, Chung MK, Fan J, Rabinowitz JD. Quantitative analysis of acetyl-CoA production in hypoxic cancer cells reveals substantial contribution from acetate. *Cancer & metabolism* 2014; **2**(1): 23.

11. Metallo CM, Gameiro PA, Bell EL, Mattaini KR, Yang J, Hiller K *et al.* Reductive glutamine metabolism by IDH1 mediates lipogenesis under hypoxia. *Nature* 2012; **481**(7381): 380.

12. Mylonis I, Sembongi H, Befani C, Liakos P, Siniossoglou S, Simos G. Hypoxia causes triglyceride accumulation by HIF-1-mediated stimulation of lipin 1 expression. *J Cell Sci* 2012; **125**(14): 3485-3493.

13. Schlaepfer IR, Nambiar DK, Ramteke A, Kumar R, Dhar D, Agarwal C *et al.* Hypoxia induces triglycerides accumulation in prostate cancer cells and extracellular vesicles supporting growth and invasiveness following reoxygenation. *Oncotarget* 2015; **6**(26): 22836.

14. Yu Y, Vidalino L, Anesi A, Macchi P, Guella G. A lipidomics investigation of the induced hypoxia stress on HeLa cells by using MS and NMR techniques. *Molecular BioSystems* 2014; **10**(4): 878-890.

15. Lisec J, Jaeger C, Zaidi N. Cancer cell lipid class homeostasis is altered under nutrient-deprivation but stable under hypoxia. *bioRxiv* 2018: 382457.

16. Gharpure KM, Pradeep S, Sans M, Rupaimoole R, Ivan C, Wu SY *et al.* FABP4 as a key determinant of metastatic potential of ovarian cancer. *Nature communications* 2018; **9**(1): 2923.

17. Du W, Zhang L, Brett-Morris A, Aguila B, Kerner J, Hoppel CL *et al.* HIF drives lipid deposition and cancer in ccRCC via repression of fatty acid metabolism. *Nature communications* 2017; **8**(1): 1769.

18. Schug ZT, Peck B, Jones DT, Zhang Q, Grosskurth S, Alam IS *et al.* Acetyl-CoA synthetase 2 promotes acetate utilization and maintains cancer cell growth under metabolic stress. *Cancer cell* 2015; **27**(1): 57-71.

19. Peck B, Schug ZT, Zhang Q, Dankworth B, Jones DT, Smethurst E *et al.* Inhibition of fatty acid desaturation is detrimental to cancer cell survival in metabolically compromised environments. *Cancer & metabolism* 2016; **4**(1): 6.

20. Roongta UV, Pabalan JG, Wang X, Ryseck R-P, Fargnoli J, Henley BJ *et al.* Cancer cell dependence on unsaturated fatty acids implicates stearoyl-CoA desaturase as a target for cancer therapy. *Molecular Cancer Research* 2011; **9**(11): 1551-1561.

21. Ackerman D, Tumanov S, Qiu B, Michalopoulou E, Spata M, Azzam A *et al.* Triglycerides Promote Lipid Homeostasis during Hypoxic Stress by Balancing Fatty Acid Saturation. *Cell reports* 2018; **24**(10): 2596-2605. e2595.

22. Zaidi N, Royaux I, Swinnen JV, Smans K. ATP citrate lyase knockdown induces growth arrest and apoptosis through different cell-and environment-dependent mechanisms. *Molecular cancer therapeutics* 2012; **11**(9): 1925-1935.

23. Daniels VW, Smans K, Royaux I, Chypre M, Swinnen JV, Zaidi N. Cancer cells differentially activate and thrive on de novo lipid synthesis pathways in a low-lipid environment. *PloS one* 2014; **9**(9): e106913; doi 10.1371/journal.pone.0106913.

24. Usman H, Ameer F, Munir R, Iqbal A, Zaid M, Hasnain S *et al.* Leukemia cells display lower levels of intracellular cholesterol irrespective of the exogenous cholesterol availability. *Clinica chimica acta; international journal of clinical chemistry* 2016; **457**: 12-17; doi 10.1016/j.cca.2016.03.015.

25. Ameer F, Munir R, Usman H, Rashid R, Shahjahan M, Hasnain S *et al.* Lipid-load in peripheral blood mononuclear cells: Impact of food-consumption, dietary-macronutrients, extracellular lipid availability and demographic factors. *Biochimie* 2017; **135**: 104-110; doi 10.1016/j.biochi.2017.01.015.

26. Bulusu V, Tumanov S, Michalopoulou E, van den Broek NJ, MacKay G, Nixon C *et al.* Acetate Recapturing by Nuclear Acetyl-CoA Synthetase 2 Prevents Loss of Histone Acetylation during Oxygen and Serum Limitation. *Cell reports* 2017; **18**(3): 647-658; doi 10.1016/j.celrep.2016.12.055.
